# People’s Experiences and Satisfaction With Telehealth During the COVID-19 Pandemic in Australia: Cross-Sectional Survey Study

**DOI:** 10.2196/24531

**Published:** 2020-12-10

**Authors:** Jennifer MJ Isautier, Tessa Copp, Julie Ayre, Erin Cvejic, Gideon Meyerowitz-Katz, Carys Batcup, Carissa Bonner, Rachael Dodd, Brooke Nickel, Kristen Pickles, Samuel Cornell, Thomas Dakin, Kirsten J McCaffery

**Affiliations:** 1 Faculty of Medicine and Health Sydney Health Literacy Lab, School of Public Health The University of Sydney Sydney Australia; 2 Population Wellbeing and Environment Research Lab School of Health and Society University of Wollongong Wollongong Australia; 3 Western Sydney Diabetes Western Sydney Local Health District Sydney Australia

**Keywords:** COVID-19, patient experience, telehealth, experience, satisfaction, telemedicine, Australia, usability, cross-sectional, survey

## Abstract

**Background:**

In response to the COVID-19 pandemic, telehealth has rapidly been adopted to deliver health care services around the world. To date, studies have not compared people’s experiences with telehealth services during the pandemic in Australia to their experiences with traditional in-person visits.

**Objective:**

This study aimed to compare participants’ perceptions of telehealth consults to their perceptions of traditional in-person visits and investigate whether people believe that telehealth services would be useful after the pandemic.

**Methods:**

A national, cross-sectional, community survey was conducted between June 5 and June 12, 2020 in Australia. In total, 1369 participants who were aged ≥18 years and lived in Australia were recruited via targeted advertisements on social media (ie, Facebook and Instagram). Participants responded to survey questions about their telehealth experience, which included a free-text response option. A generalized linear model was used to estimate the adjusted relative risks of having a poorer telehealth experience than a traditional in-person visit experience. Content analysis was performed to determine the reasons why telehealth experiences were worse than traditional in-person visit experiences.

**Results:**

Of the 596 telehealth users, the majority of respondents (n=369, 61.9%) stated that their telehealth experience was “just as good as” or “better than” their traditional in-person medical appointment experience. On average, respondents perceived that telehealth would be moderately useful to very useful for medical appointments after the COVID-19 pandemic ends (mean 3.67, SD 1.1). Being male (*P*=.007), having a history of both depression and anxiety (*P*=.016), and lower patient activation scores (ie, individuals’ willingness to take on the role of managing their health/health care) (*P*=.036) were significantly associated with a poor telehealth experience. In total, 6 overarching themes were identified from free-text responses for why participants’ telehealth experiences were poorer than their traditional in-person medical appointment experiences, as follows: communication is not as effective, limitations with technology, issues with obtaining prescriptions and pathology results, reduced confidence in their doctor, additional burden for complex care, and inability to be physically examined.

**Conclusions:**

Based on our sample’s responses, telehealth appointment experiences were comparable to traditional in-person medical appointment experiences. Telehealth may be worthwhile as a mode of health care delivery while the pandemic continues, and it may continue to be worthwhile after the pandemic.

## Introduction

The COVID-19 outbreak was officially declared a pandemic by the World Health Organization on March 11, 2020. To help minimize the spread of COVID-19, health care systems have rapidly adopted alternative models for health care delivery, including telehealth services [1]. This type of health care delivery minimizes the spread of the virus by providing health care services without the need for close contact, thereby reducing the risk of exposure to COVID-19 for both patients and clinicians.

In response to the COVID-19 pandemic, the Australian Government introduced a temporary telehealth scheme on March 30, 2020 to enable subsidized access to health care services that are provided via telephone or videoconferencing [2]. Prior to the pandemic, telehealth consultations were restricted to rural and remote communities. This new scheme has allowed all medical appointments with a variety of health professionals to be conducted via telehealth, regardless of rurality. As a result of this scheme, telehealth consults have accounted for 36% of all services provided in April 2020, whereas telehealth consults conducted before the pandemic only accounted for 1.3% [3,4]. At the end of April 2020, a nationally representative survey of 1022 people conducted by the Australian Bureau of Statistics reported that 1 in 6 people (17%) used a telehealth service, women were almost twice as likely as men to use telehealth services (22% vs 12%), and persons with a chronic or mental health condition were twice as likely to have used a telehealth service compared to those without such conditions (25% vs 13%). However, 1 in 10 people (10%) reported to have a general practitioner or health professional appointment cancelled or postponed in the last 4 weeks because of the COVID-19 pandemic [5].

Cancelling or postponing appointments is concerning because reduced health care during pandemics has been associated with poor health outcomes, as observed during the Ebola virus outbreak and Severe acute respiratory syndrome epidemic [6,7]. The increased uptake of telehealth services and increased number of people cancelling or postponing medical appointments warrants further investigation to better understand people’s experiences and satisfaction with accessing telehealth services during the COVID-19 pandemic. This is particularly necessary, given the long-term outlook of the COVID-19 pandemic; although several health services have returned to normal, continuing outbreaks may deter patients from accessing in-person care for some time [8].

Despite the growth of telehealth, no studies have compared people’s experiences with telehealth services during the COVID-19 pandemic in Australia to people’s experiences with traditional in-person visits. We investigated a sample of Australians and their experiences with telehealth during the COVID-19 pandemic. Our aims were to compare participants’ perceptions of telehealth consults to their perceptions of traditional in-person visits and investigate whether people believe that telehealth services would be useful after the pandemic. Furthermore, we investigated the sociodemographic and health-related factors associated with negative telehealth experiences.

## Methods

### Recruitment

The data used in this study are from a prospective, longitudinal, national survey that launched in April 2020 and explored variations in people’s understanding of, attitude toward, and uptake of COVID-19 health advice during the 2020 pandemic [9]. Herein, we report on data from a survey wave conducted over a 1-week period (ie, June 5 to June 12, 2020) in Australia. Data were obtained using the Qualtrics online platform. Participants who were aged ≥18 years, could read and understand English, and resided in Australia were recruited via paid targeted advertisements on social media (ie, Facebook and Instagram). More details on recruitment are provided in the McCaffery et al study [9]. Participants were given the opportunity to enter a prize draw for the chance to win 1 of 10 Aus $20 (US $14.62) gift cards upon completion of the survey. This study was approved by The University of Sydney Human Research Ethics Committee (2020/212).

### Measures

Sociodemographic variables, including age, gender, and educational status, were collected, along with data on self-reported chronic diseases and overall health. We assessed health literacy using the Newest Vital Sign [10] and digital health literacy using the eHealth Literacy Scale [11]. The Consumer Health Activation Index [12] was used to determine patient activation (ie, individuals’ willingness to take on the role of managing their health and health care). The remoteness and socioeconomic status of participants’ places of residence were derived from participants’ postcodes [13]. Participants were asked to indicate whether they had used telehealth services. If so, they were then asked how telehealth services compared to traditional in-person visits, whether they experienced any barriers to using telehealth services, and whether they cancelled or postponed an appointment with a health professional (Textbox 1).

**Survey items and scoring scale on telehealth.**

**Telehealth usage**

- Since the COVID-19 restrictions started, have you had a telemedicine/telehealth appointment (appointment with your health provider by video or phone instead of an in-person appointment)? (Response options: Yes/No)
- How many telehealth appointments have you had? (Response: Numerical [free-text])
- Was/were your telemedicine/telehealth visit(s) done by: (Response options: Telephone/Videoconference/Both)

**Comparison between telehealth and traditional in-person visits**

- How did your telemedicine/telehealth visit compare to a traditional in-person medical visit? (Response options: Better than a traditional visit/Just as good as a traditional visit/Worse than a traditional visit/Not sure)
- If, telemedicine/telehealth was worse, please tell us why. (Response: Free text)

**Interest in telehealth after COVID-19**

- How useful do you think it will be to have medical appointments with telemedicine/telehealth after the COVID-19 emergency is over? (Response scale: 1-5, indicating not at all to extremely)

**Cancellation of in-person appointments**

- Have you cancelled or postponed an appointment with a health professional in the last 4 weeks because of COVID-19? (Response options: Yes/No)
- Why? (Response options: Concerned about the cost/I am isolating due to COVID-19 symptoms or risk/I was worried about travelling on public transport because of the COVID-19 risk/I did not want to go to a health or hospital clinic because of concerns about catching COVID-19 there/Too busy Other [please tell us])
- Did you feel you needed to see a health professional in person in the last 4 weeks but chose not to go? (Response options: Yes/No)
- Why? (Response options: Concerned about the cost/I am isolating due to COVID-19 symptoms or risk/I was worried about travelling on public transport because of the COVID-19 risk/I did not want to go to a health or hospital clinic because of concerns about catching COVID-19 there/Too busy/Other [please tell us])

**Barriers to telehealth**

- Have you needed to access a telehealth service in the last 4 weeks but could not? (Response options: Yes/No)
- What was the main reason that you could not access a telehealth service in the last 4 weeks? (Response options: Telehealth not available from general practitioner or other health professional/Do not have internet/I am not able to use the internet/Dislike or fear of the service/Appointment not available when required/Other [please detail])

### Statistical Analysis

Quantitative data were analyzed using Stata/IC v16 (StataCorp LLC). Descriptive statistics were analyzed to obtain sample characteristics and summarize participants’ telehealth experiences since COVID-19 restrictions commenced. A generalized linear model using a modified Poisson approach (ie, log link function with robust standard errors) was used to estimate adjusted relative risks with 95% confidence intervals for having a poorer telehealth experience than a traditional in-person medical visit experience based on various sociodemographic and health-related factors. A 2-tailed independent samples *t* test was used to compare the perceived usefulness of telehealth medical appointments once the COVID-19 emergency ends between participants who rated their telehealth experience as worse than their in-person medical visit experience and those who rated their telehealth experience as the same as or better than their in-person medical visit experience. The statistical significance for these exploratory analyses was set at *P*<.05 (2-tailed).

Qualitative data were analyzed using content analysis [14], which combines both qualitative and quantitative methods and allows for both the frequency of categories and the content to be reported. JI and TC familiarized themselves with the content and generated a list of recurring themes; these were discussed with and checked by an additional researcher (JA). JI and TC then applied the final coding framework to all the data. The level of agreement was tested using the Cohen kappa, which indicated substantial agreement (κ=0.76) [15]. Discrepancies were discussed until a consensus was obtained. Descriptive statistics were provided to summarize the frequency of each code.

## Results

Of the 1369 respondents who completed the June survey, 596 (43.5%) reported using telehealth services since the start of the pandemic. Respondents who used telehealth services were slightly older; more likely to be female; had higher levels of education; had a greater prevalence of chronic health conditions, including a history of mental health conditions; and had poorer self-reported general health compared to those who did not use telehealth services. Sample characteristics are summarized in Table 1.

**Table 1:** Descriptive characteristics of our sample sorted by participants’ use of telehealth services during the COVID-19 lockdown period.

Variable		Accessed telehealth services (n=596)	Did not access telehealth services (n=773)	Overall (N=1369)
Age in years, mean (SD)		46.2 (16.1)	43.6 (17.0)	44.7 (16.7)
**Age group (years), n (%)**				
	18-25	76 (12.8)	156 (20.2)	232 (16.9)
	26-40	166 (27.9)	206 (26.6)	372 (27.2)
	41-55	152 (25.5)	192 (24.8)	344 (25.1)
	56-90	202 (33.9)	219 (28.3)	421 (30.8)
**Gender, n (%)**				
	Male	146 (24.5)	287 (37.1)	433 (31.6)
	Female	433 (72.7)	478 (61.8)	911 (66.5)
	Other/prefer not to say	17 (2.9)	8 (1)	25 (1.8)
**Highest level of education completed, n (%)**				
	High school or less	68 (11.4)	130 (16.8)	198 (14.5)
	Certificate I-IV	67 (11.2)	73 (9.4)	140 (10.2)
	University education	461 (77.3)	570 (73.7)	1031 (75.3)
**Number of chronic health conditions^a^, n (%)**				
	0	239 (40.1)	436 (56.4)	675 (49.3)
	1	188 (31.5)	220 (28.5)	408 (29.8)
	≥2	169 (28.4)	117 (15.1)	286 (20.9)
**Mental health history, n (%)**				
	Depression	278 (46.6)	193 (25.0)	471 (34.4)
	Anxiety	302 (50.7)	232 (30)	534 (39)
**Self-reported general health, n (%)**				
	Poor	37 (6.2)	9 (1.2)	46 (3.4)
	Fair	111 (18.6)	76 (9.8)	187 (13.7)
	Good	226 (37.9)	237 (30.7)	463 (33.8)
	Very Good	172 (28.9)	321 (41.5)	493 (36)
	Excellent	50 (8.4)	130 (16.8)	180 (13.1)
Socioeconomic status, mean IRSAD^b^ quintile (SD)		3.7 (1.4)	3.7 (1.4)	3.7 (1.4)
**Remoteness, n (%)**				
	Major cities	438 (73.5)	589 (76.2)	1027 (75)
	Other	158 (26.5)	184 (23.8)	342 (25)
Adequate health literacy^c^, n (%)		505 (90.3)	665 (91.7)	1170 (91.1)
eHealth literacy^d^, mean (SD)		4.2 (0.7)	4.1 (0.7)	4.2 (0.7)
Patient activation^e^, mean (SD)		74.7 (13.2)	75.0 (13.4)	74.9 (13.3)
Cancelled/postponed an appointment^f^, n (%)		147 (24.7)	125 (16.2)	272 (19.9)
Chose not to see a health professional^g^, n (%)		115 (19.3)	104 (13.5)	219 (16)
Could not access telehealth services^h^, n (%)		12 (2)	7 (0.9)	19 (1.4)

^a^Chronic health conditions included respiratory disease, asthma, chronic obstructive pulmonary disease, hypertension, cancer, heart disease, stroke, and diabetes.

^b^IRSAD: Index of Relative Socio-Economic Advantage and Disadvantage. In the IRSAD quintile [13], a score of 1 represents most disadvantaged and a score of 5 represents most advantaged.

^c^Health literacy was assessed using the Newest Vital Sign [10]. Data were missing for 85 (6.2%) participants percent due to technical errors with the Qualtrics online platform.

^d^eHealth [11] literacy was measured on a 5-point Likert scale. A higher score reflects a higher level of eHealth literacy.

^e^Results are based on the Consumer Health Activation Index [12]. A score of 0-79 indicates low activation, 80-94 indicates moderate activation, and 95-100 indicates high activation.

^f^Respondents who cancelled/postponed an appointment in the last 4 weeks because of COVID-19.

^g^Respondents who felt the need to see a health professional in the last 4 weeks, but chose not to.

^h^Respondents who needed access to a telehealth service in the last 4 weeks, but could not.

### Cancellation of In-Person Appointments

Of the 1369 total respondents, 272 (19.9%) cancelled or postponed an in-person appointment with a health professional. The reasons for cancelling appointments were as following: concerns about catching COVID-19 at a clinic or hospital (n=85, 31.3%), isolating due to COVID-19 symptoms or risks (n=31, 11.4%), concerns about travelling via public transport (n=21, 7.7%), feeling too busy (n=20, 7.4%), cost (n=9, 3.3%), and other reasons (n=106, 39%). Less common reasons for cancelling or postponing an in-person appointment included the following: border closures, postponed elective surgery, and the appointment seemed nonessential. Furthermore, 219 (16%) respondents felt that they needed to see a health professional in-person in the last 4 weeks, but chose not to go due to the following reasons: concerns about catching COVID-19 at a clinic or hospital (n=72, 32.9%), feeling too busy (n=37, 16.9%), isolating due to COVID-19 symptoms or risks (n=18, 8.2%), concerns about travelling via public transport (n=13, 5.9%), other reasons (n=67, 30.6%). Less common reasons listed for choosing not to see a health professional included the following: only telehealth services were available, limited in-person appointment availability, and felt that seeing a health professional was too complicated.

### Telehealth Experiences

The characteristics of telehealth users’ experiences are shown in Table 2. Of the 596 respondents who used telehealth services, over half (n=326, 54.7%) reported having more than 1 telehealth appointment, of which most were conducted by telephone (n=427, 71.6%). The majority of respondents (n=369, 61.9%) stated that their telehealth experience was “just as good as” or “better than” their traditional in-person medical visit experience. On average, respondents perceived telehealth as moderately useful to very useful for medical appointments after the COVID-19 pandemic ends (mean 3.67, SD 1.1). Individuals who responded that their telehealth experience was worse than their traditional in-person medical visit experience (n=205, 34.4%) also rated the usefulness of telehealth after the COVID-19 emergency ends significantly lower than those whose telehealth experience was “just as good as” or “better than” their in-person visit experience (mean 2.86 vs mean 4.17; difference: mean 1.31; 95% CI 1.14-1.47; t_572_=15.62; *P*<.001).

**Table 2:** Characteristics of telehealth users’ experience (n=596).

Variable		Summary value, n (%)
**Number of telehealth appointments**		
	1	270 (45.3)
	2	157 (26.3)
	≥3	169 (28.4)
**Mode of telehealth delivery**		
	Telephone	427 (71.6)
	Videoconference	84 (14.1)
	Both	85 (14.3)
**Telehealth visit compared to traditional in-person medical visit**		
	Better	49 (8.2)
	Just as good	320 (53.7)
	Worse	205 (34.4)
	Unsure	22 (3.7)

### Factors Associated With a Poor Telehealth Experience

The results of the multivariable analysis that explored factors associated with a poorer telehealth experience than an in-person appointment experience are displayed in Table 3. Being male (*P*=.007), having a history of both depression and anxiety (*P*=.04), and having a low patient activation score (*P*=.04) were associated with a poorer telehealth experience, after controlling for all other variables in the model.

**Table 3:** Multivariable^a^ analysis of factors associated with a poorer^b^ telehealth experience than an in-person appointment experience (n=574). Adjusted relative risks of <1 indicate a reduced risk of reporting a poorer telehealth experience relative to the reference group.

Variable		Adjusted relative risk (95% CI)	*P* value
**Age (years)^c^**			.27
	18-25	0.98 (0.66-1.47)	.94
	26-40	1.09 (0.80-1.49)	.57
	56-90	1.32 (0.97-1.80)	.08
**Gender^d^**			.01
	Female	0.73 (0.58-0.92)	.007
	Other/prefer not to say	0.52 (0.24-1.14)	.11
**Highest level of education completed^e^**			.96
	Certificate I-IV	1.06 (0.67-1.67)	.80
	University education	1.01 (0.69-1.48)	.96
**Number of chronic health conditions^f^**			.26
	1	0.88 (0.68-1.15)	.35
	≥2	0.78 (0.58-1.05)	.11
**Mental health history^g^**			.054
	Either depression or anxiety	1.27 (0.92-1.75)	.14
	Both depression and anxiety	1.42 (1.07-1.89)	.016
Socioeconomic status (per IRSAD^h^ quintile)		1.06 (0.97-1.16)	.20
eHealth literacy (per unit)		1.02 (0.84-1.23)	.84
Patient activation (per 10-unit increase)		0.91 (0.82-0.99)	.036
**Telehealth delivery mode^i^**			.23
	Videoconference	1.28 (0.96-1.70)	.09
	Both telephone and videoconference	1.09 (0.79-1.51)	.60

^a^The analysis also controlled for the number of telehealth visits since lockdown.

^b^A poorer outcome was defined as respondents rating their telehealth experience as worse (compared to “just as good as” or “better than”) than their traditional in-person medical visit experience. Individuals who responded with “unsure” (22/596, 3.7%) were excluded from the analysis.

^c^Respondents aged 41-55 years were used as a reference.

^d^Male respondents were used as a reference.

^e^Respondents who completed a high school education or less were used as a reference.

^f^Respondents who did not have a chronic health condition were used as a reference.

^g^Respondents who did not have a history of mental health conditions were used as a reference.

^h^IRSAD: Index of Relative Socio-Economic Advantage and Disadvantage.

^i^Respondents who had telehealth visits via telephone were used as a reference.

### Reasons Provided for Why Telehealth Experiences Were Worse Than Traditional In-Person Visit Experiences

In total, the following 6 overarching themes regarding telehealth services (Table 4) emerged from the free-text data: (1) communication is not as effective as face-to-face visits, (2) limitations with technology, (3) issues with obtaining prescriptions and pathology results, (4) reduced confidence in the doctor, (5) additional burden for complex care, and (6) inability to be physically examined. Of the 221 respondents who provided a free-text response for why their telehealth experiences were worse than their traditional in-person visit experiences, the majority received telehealth services via telephone (n=149, 67.4%), whereas comparatively fewer respondents received telehealth services via videoconference (n=37, 16.7%) or a combination of both (n=35, 15.8%). However, no substantial differences in the overarching themes were observed between these groups. Overall, the most recurrent theme was that communication was not as effective as traditional in-person visits due to the lack of visual cues, eye contact, and body language.

**Table 4:** Reasons provided by 221 respondents for telehealth visits being worse than traditional in-person medical visits, along with the frequency of overarching themes and subthemes with example quotes^a^.

Code description			Example		Value, n (%)
**­Communication is not as effective as face-to-face visits**					
	Lacks visual cues, eye contact, body language, and visual feedback; face-to-face visits are preferred	“The subtle facial expressions eye contact and body language are not the same”		54 (24.4)	
	Less personal, less natural/comfortable, more awkward	“Difficult to establish rapport”		46 (20.8)	
	Less effective; communication is not as good, less helpful, and harder/more difficult	“Communication on the phone is less effective”		45 (20.4)	
	Face-to-face interaction is needed for mental health appointments	“I feel a big part of effective mental health care involves face-to-face conversation”		19 (8.6)	
	More anxiety provoking for some	“Phone call and videos make me extremely anxious”		5 (2.3)	
	Less privacy	“Lack of privacy”		3 (1.4)	
**The inability to be physically examined**					
	Physical exam is not possible	“Could not have a physical exam done”		60 (27.1)	
	Tests could not be performed	“Blood pressure not taken”		17 (7.7)	
**Limitations with technology**					
	Technology issues, including poor connection, bad reception, poor audio quality, and Zoom calls dropping out	“Due to audio quality I was not able to get names of chemotherapy drugs correctly - so when I tried to look up info later I couldn’t until I was able to get info from Breast care nurse so this added to days of anxiety due to lack of info over weekend and when that staff member on leave.”		20 (9)	
	Poor quality connection led to poor quality conversation	“Harder to communicate due to tech difficulties, lag issues”		6 (2.7)	
**Issues with obtaining prescriptions and pathology results**					
	Harder to obtain prescriptions	“I had to wait for scripts to be emailed to the pharmacy, then one was missing, which I could have seen at the time had I received them in person.”		10 (4.5)	
	Increased wait time/delayed access	“If you need a script or referral, you have to make a separate trip to go get the paper”		7 (3.2)	
	Unable to access pathology results	“Getting blood tests has become more difficult.”		2 (0.9)	
**Reduced confidence in doctor/health professional**					
	Not as comprehensive or thorough	“Not as comprehensive and thorough”		25 (11.3)	
	Time pressure	“Felt rushed”		18 (8.1)	
	Lack of confidence in assessment/ diagnosis	“Less trust that the diagnosis is accurate”		11 (5)	
**Additional burden for complex care**					
	Face-to-face visit required due to complex issues	“I had to go in for a face-face consult because the medical issues could not be diagnosed”		15 (6.8)	
	Delays due to complex issues	“More complex issues have been delayed until we can do face-to-face”		5 (2.3)	
	Added burden of having 2 consults	“In both instances after having the Telehealth calls, I had to go in for a face to face consults because the issues could not be diagnosed over the phone”		4 (1.8)	

^a^Full text could have more than 1 theme applied.

### Barriers to Telehealth

Of the 1369 total respondents, 19 (1.4%) reported that they were not able to access a telehealth service. The barriers reported by these 19 respondents were as follows: telehealth services were not available from their general practitioner or health professional (n=4, 21.1%), they did not have internet (n=2, 10.5%), the appointment was not available when required (n=8, 42.1%), and using telehealth services felt too complicated (n=5, 26.3%).

## Discussion

### Principal Results

Our findings showed that more than half of the respondents (n=369, 61.9%) stated that their telehealth experience was “just as good as” or “better than” their traditional in-person medical care experience. This is encouraging, considering that the community transmission of COVID-19 across Australia may continue to persist for some time. On average, respondents perceived that telehealth would be moderately useful to very useful for medical appointments after the COVID-19 pandemic. This suggests that telehealth may be a viable long-term option for health care delivery. Our findings are consistent with another survey, which reported that 85% of older Australians found their telehealth experiences to be similar or better than their experiences with face-to-face consults [16]. Furthermore, we found that telehealth delivery modality (ie, telephone and video) was not associated with having a poorer telehealth experience than an in-person appointment experience (*P*=.23). This is consistent with other studies that showed telephone and videoconferencing were comparable in terms of patient satisfaction [17]. It is perhaps unsurprising that respondents who rated their telehealth experiences as worse than their traditional in-person visit experiences were less likely to perceive that telehealth would be useful after the COVID-19 pandemic. Such respondents were more likely to be male, have lower patient activation scores (ie, individuals’ willingness to take on the role of managing their health and health care), and have a history of both depression and anxiety (Table 3). This last observation is also supported by the content analysis, which highlighted that several participants preferred in-person to telehealth visits for mental health appointments.

### Limitations

While the study sample was large and diverse, it was not statistically representative of the Australian population. Our sample was recruited via social media, which was likely the reason why our sample consisted of a higher proportion of females, higher level of education, and potentially higher levels of digital literacy than the general population [18]. A further limitation of this study was that we did not know the percentage of people who attended in-person consults during the study period, nor did we know whether those who did not access telehealth services required a health appointment or attended an in-person consult instead. In addition, our survey did not collect any information on the type of telehealth services that participants attended (eg, allied health and specialist health services). Future studies should investigate whether the factors associated with a poorer telehealth experience are similar across the models of health care delivery. Furthermore, future surveys should compare people’s experiences with telehealth to people’s experiences with traditional in-person visits based on the type of health service provided (eg, general practitioner, specialist, and allied health services) and determine the impact of different health service modalities and types on health outcomes.

### Comparison With Prior Work

Our findings suggest that for some, telehealth was perceived as less effective for delivering mental health services. This is concerning, as mental health problems, such as depression and anxiety, were at least twice as prevalent during the first month of the COVID-19 pandemic in Australia compared to before the pandemic [19], and this problem is only expected to grow [20]. Our findings are also similar to previous research on telehealth and mental health [21], which is concerning, as negative experiences with telehealth may result in no mental health care for patients if face-to-face services are unavailable.

The most common theme for why respondents perceived telehealth to be worse than in-person medical care was less effective communication. This issue can be addressed by encouraging the use of established strategies for improving communication between health professionals and patients. For example, the teach-back method, which is also known as the show-me method or closing-the-loop method, has been shown to increase people’s understanding of health information by asking patients to repeat health information in their own words [22]. In addition, providing a written lay summary of the visit via a patient letter or patient portal may improve patients’ telehealth experiences. Patients have reported improved patient-provider communication as a result of using a patient portal [23]. Other approaches for addressing this issue may include online education or mobile apps, which have both been used to enhance patients’ understanding of content and improve health care services [24-27]. However, it should be noted that the Australian government is currently fast-tracking electronic prescribing, which may improve communication between general practitioners, patients, and pharmacists [28]. Electronic prescribing allows for the easy electronic sharing of prescriptions, thereby eliminating issues related to the challenge of obtaining prescriptions from telehealth appointments.

The following issues regarding access to telehealth services were also identified in our study: physical examination was not possible, people were less confident in their doctor or health care professional during telehealth visits, and additional burden was experienced for complex health conditions. These issues can be addressed by setting clear expectations for telehealth when scheduling appointments and explaining which types of appointments are suitable for telehealth. For example, when appointments are scheduled, patients should be notified that additional in-person consults may be required depending on the complexity of their medical appointment and that telehealth services may not be appropriate for mental health issues. In addition, videoconferencing could be offered, as it may allow for more reliable visual assessments and more accurate diagnoses [17]. Overall, in order to improve patients’ experiences with telehealth, strategies should be implemented to ensure that patients are aware of what to expect from telehealth appointments.

It is important to note that, in our study, 19.9% (272/1369) of respondents cancelled or postponed an in-person health appointment. The main reason for cancelling appointments was concern about catching COVID-19 at health or hospital clinics. Our study found a higher proportion of people who cancelled or postponed a health appointment than a survey conducted by the Australian Bureau of Statistics (11%) [5]. Similarly, a study of 151 women with breast cancer conducted in Israel found that 31% of people cancelled a health appointment, with the most common reason being the fear of contracting COVID-19 [29]. This is worrying, as continuing outbreaks may deter patients from accessing essential in-person medical care for some time. Therefore, our results suggest that telehealth services should continue to be offered while the community transmission of COVID-19 persists. Future studies should investigate whether patients who cancel or postpone health appointments are seeking telehealth services and monitor the long-term impact of the use of health services on health outcomes.

### Future Directions and Considerations of Telehealth

It is worth noting that the temporary telehealth scheme is scheduled to end on March 31, 2021 [30]. It is unclear as to what degree and for whom telehealth services will be subsidized after this date. Telehealth has the potential to reduce inequality by making health care services more accessible. However, in order for telehealth to be an effective public health service, it should be widely available and affordable. Our findings showed participants’ willingness to use telehealth services after the pandemic. However, we did not investigate whether people would engage with telehealth services if these services were no longer subsidized. It is reasonable to expect that people’s willingness to use telehealth services may change, depending on funding reforms. Future research should continue to investigate patients’ attitudes toward telehealth as policies change over time. 

Our study provides insight into patients’ perceptions of telehealth services. However, we did not investigate health professionals’ perspectives and perceptions, including their experiences with telehealth, confidence to deliver telehealth, and willingness to continue to provide telehealth services beyond the pandemic. Further studies should investigate both patients’ and health professionals’ attitudes toward and experiences with telehealth, as they are both important voices in discussions about the future of telehealth in Australia.

### Conclusions

Overall, we found that respondents’ telehealth experiences were comparable to their experiences with traditional in-person visits. We identified the most common reasons for a poor experience with telehealth and provided strategies for improving the experience of telehealth users. In light of our results, telehealth may be worthwhile as a mode of health care delivery while the pandemic continues, and it may be worthwhile beyond the pandemic. However, studies on a broader, more representative sample of the Australian population are warranted.
